# Bacterial metabolites trimethylamine N-oxide and butyrate as surrogates of small intestinal bacterial overgrowth in patients with a recent decompensated heart failure

**DOI:** 10.1038/s41598-021-85527-5

**Published:** 2021-03-17

**Authors:** Anna Mollar, Vannina G. Marrachelli, Eduardo Núñez, Daniel Monleon, Vicent Bodí, Juan Sanchis, David Navarro, Julio Núñez

**Affiliations:** 1 Cardiology Department, Hospital Clínico Universitario, INCLIVA. Universitat de València, Avda. Blasco Ibáñez 17, 46010 Valencia, Spain; 2 CIBER Cardiovascular, Madrid, Spain; 3 Metabolomic and Molecular Image Lab, Health Research Institute, INCLIVA, Valencia, Spain; 4 Physiology Department, Universitat de Valencia, Valencia, Spain; 5 Microbiology Department. Hospital Clínico Universitario, INCLIVA, Universitat de València, Valencia, Spain

**Keywords:** Biomarkers, Cardiology, Risk factors

## Abstract

In patients with heart failure (HF), the exhaled concentrations of hydrogen after a breath test—a non-invasive assessment of small intestinal overgrowth- has been related to HF severity and higher risk of adverse outcomes. Indeed, two intestinal bacterial metabolites—blood Trimethylamine N-Oxide (TMAO) and butyrate—have been related to a worse prognosis in HF. However, the relationship between the exhaled concentrations of hydrogen after a breath test and these two metabolites remains unknown. Thus, in this post-hoc analysis, we sought to evaluate whether these two metabolites are associated with the exhaled concentrations of hydrogen after a breath test in patients with a recent admission for HF. We included 60 patients with a recent hospitalization for HF. Cumulative hydrogen over time was integrated into a single measurement by the area under the concentration curve (AUC-H2). A linear regression multivariable analysis was used to evaluate the associations. A 2-sided p-value < 0.05 was considered to be statistically significant. The median (p25–p75) amino-terminal pro-brain natriuretic peptide, AUC-H2, TMAO, and Butyrate were 4789 pg/ml (1956–11149), 1615 (700–2585), 0.68 (0.42–1.12), and 0.22 ± 13, respectively. After multivariate adjustment, TMAO and butyrate were significantly associated with AUC-H2 (p = 0.027 and p = 0.009, respectively). For TMAO, this association was positive and for butyrate, negative. Bacterial-origin metabolites TMAO and Butyrate were independently related to AUC-H2 in patients with a recent hospitalization for acute HF.

## Introduction

There is increasing evidence about the role of gut disturbances in heart failure (HF)^1,2^. HF has been linked to intestinal bacterial overgrowth, low bacterial richness, increased intestinal permeability, dysbiosis, and the presence of bacterial metabolites in the bloodstream^1–3^. Additionally, an altered intestinal microbiome has been pointed out as a potential player in HF progression^4^. In recent work, we found that the exhaled concentration of hydrogen after a lactulose breath test—a surrogate of small intestinal overgrowth (SIBO)—was associated with greater inflammatory activity, HF severity, and a higher risk of adverse outcomes in 102 patients with HF^5^. Recent evidence also supports a crucial role in cardiovascular diseases for some gut bacterial metabolites^6^. For instance, trimethylamine N-Oxide (TMAO) was associated with a strong pro-atherosclerotic activity^7^ and with a higher risk of adverse cardiovascular events in different CV-scenarios, including HF^8^. In contrast, butyrate, a short-chain fatty acid, shows anti-inflammatory properties in the intestinal mucosa by inducing colonic regulatory T cells and may control local dysbiosis, inflammation, and permeability^2,3^. In the HF setting, some studies have reported a reduction of butyrate-producing bacteria in some patients, together with the activation of the immune system^2^.

Aiming to evaluate whether TMAO and butyrate share a common pathophysiological link with SIBO, in this post-hoc analysis, we sought to evaluate whether serum samples of TMAO and butyrate are associated with the exhaled concentration of hydrogen after a lactulose test in a cohort of patients with a recent HF decompensation.

## Material and methods

Study population and study protocol were previously described elsewhere^5^. In this sub-analysis, we only included patients with a recent hospitalization for acute HF (within the previous 30 days) in which metabolite samples were available (82.2% of the acute HF patients included in the original work). Serum samples and lactulose breath tests were performed the same day in an outpatient visit. Lactulose breath test consisted of measuring the amount of hydrogen principally produced by intestinal bacteria after ingestion of a glucose-substrate (lactulose) in one breath over repeated times (every 20 min, six times). The highest concentrations of hydrogen are presumed to be associated with bacterial overgrowth in the small intestine, which presents a low number of bacteria in physiological conditions^9^. Over time, these cumulative hydrogen measurements were integrated into a single measure by the area under the concentration curve (AUC-H2) using the trapezoid method^10,11^. A positive result of SIBO was defined by a rise of ≥ 20 ppm in 90 min (after baseline) or by baseline levels of hydrogen > 20 ppm. Serum TMAO and butyrate were measured using a 600 MHz NMR Bruker spectrometer. Interleukin-1β, interleukin-6, interleukin-10, and tumor necrosis factor-α (TNF-α) were measured from frozen samples using a commercial assay [HSCYTMAG-60SK (Milliplex High Sensitivity Human Cytokine Magnetic Panel)]. A comprehensive transthoracic echocardiographic examination was performed using commercially available systems (iE33 and EPIQ Philips, MA, USA). All methods were carried out according to relevant guidelines and regulations. All experimental protocols were approved by the ethics and research committee of the Hospital Clínico Universitario of Valencia. Informed consent was obtained from all subjects.

Continuous variables are expressed as mean ± standard deviation (SD) or median (p25–p75) when appropriate. Discrete variables are summarized as percentages. Linear regression multivariable analyses were used to evaluate the association among TMAO and butyrate with AUC-H2. A first multivariate model was performed, adjusting for age and sex (Model 1). A second final multivariate analysis was performed, adjusting for well-recognized variables associated with HF-severity and those previously related to both metabolite disturbances in HF (Model 2). The model's predictive ability of this second model was assessed by the coefficient of determination (R^2^). Thus, the criteria for variables selection for both models were mostly based on prior knowledge/biological plausibility, independent of the p-value. All variables included in Table 1 were tested. During the selection process—backward selection—the linearity assumption for continuous variables were tested and transformed with fractional polynomials as appropriate. The final multivariate model 2 included the following covariates: age, sex, systolic blood pressure (SBP), left ventricular ejection fraction (LVEF), glomerular filtration rate (GFR), hemoglobin, interleukin-1β, and interleukin-6. A 2-sided p-value < 0.05 was considered to be statistically significant for all analyses. All analyses were performed using STATA 15.1.

**Table 1:** Baseline characteristics.

Variables	Study population, n = 60
**Demographics and medical history**	
Age, years	74 (70–78)
Male, n (%)	45 (75)
Hypertension, n (%)	50 (83)
NYHA III–IV, n (%)	28 (47)
Diabetes mellitus, n (%)	32 (53)
Dyslipidemia, n (%)	47 (78)
Ischemic etiology, n (%)	25 (42)
Valvular disease, n (%)	26 (42)
Charlson's index	2 (1–3)
Renal failure, n (%)	32 (53)
Peripheral edema, n (%)	49 (65)
Prior heart failure hospitalization, n (%)	35 (58)
Admitted for heart failure, n (%)	57 (95)
**Electrocardiogram and vital signs**	
Heart rate, bpm	80 (66–94)
SBP, mmHg	125 (115–144)
DBP, mmHg	68 (60–80)
QRS > 120 ms, n (%)	25 (41)
Atrial fibrillation, n (%)	36 (60)
**Echocardiography**	
LVEF, %	44.1 ± 16.6
LVEF < 40, %	27 (45)
LVEF 41–49, %	8 ( 13)
LVEF ≥ 50, %	25 (42)
sPAP, mm	43.6 (14.8)
TAPSE, mm	17.4 (3.9)
**Laboratory tests**	
Hemoglobin, mg/dL	12 ± 1.8
Sodium, mEq/L	140 ± 3.2
Potassium, mEq/L	4.1 ± 0.6
Creatinine, mg/dL	1.4 (1.04–2.1)
eGFR, mL/min/1.73 m^2^	46.3 (31.6–69.3)
NT-proBNP, pg/mL	4789 (1956–11149)
Interleukin-1β	3.2 (3.0–3.8)
Interleukin-6	6.9 (3.5–20.3)
TNF-α	12.7 (8.9–20.6)
Interelukin-10	2.3 (1.6–7.0)
Trimetilamine N-oxide	0.68 (0.42–1.12)
Butyrate	0.22 ± 13
SIBO, n (%)	36 (63)
AUC-H2	1615 (700–2585)
**Pharmacological treatment**	
ACEI/ARB, n (%)	25 (60)
Beta-blockers, n (%)	40 (67)
Furosemide equivalent dose, mg/day	80 (60–120)

*There are not missing values in the variables listed in this table.

Continuous variable are expressed as median (percentile 25–percentile 75) or mean ± standard deviation.

*NYHA* New york heart association class, *SBP* systolic blood pressure, *DBP* diastolic blood pressure, *LVEF* left ventricular ejection fraction, *sPAP* systolic arterial pulmonary pressure, *TAPSE* tricuspid annular plane systolic excursion, *NT-proBNP* amino-terminal pro-brain natriuretic peptide, *eGFR* estimated glomerular filtration rate, *TNF-α* tumor necrosis factor-α, *AUC-H2* area under the curve of hydrogen, *SIBO* small intestinal bacterial overgrowth, *ACEI* angiotensin converting enzyme inhibitors, *ARB* angiotensin II receptor blockers.

## Results

The median (p25–p75) age of the study population was 74 (70–78) years, and 75% were males. The proportion of patients with reduced (< 40%), preserved (≥ 50%), and midrange (41–49%) LVEF were 45%, 42%, and 13%, respectively. Ischemic was the most frequent etiology (42%). NYHA III–IV and peripheral edemas rates were 47% and 65%, respectively. Median (p25-p75) GFR and amino-terminal pro-brain natriuretic peptide (NT-proBNP), were 46.3 (31.6–69.3) and 4789 pg/ml (1956–11149), respectively. SIBO was positive in 27 patients (45%), and the median (percentile 25%-percentile 75%) value of AUC-H2 was 1615 (700–2585). The median of TMAO and the mean of butyrate results were 0.68 (0.42–1.12) and 0.22 ± 13. All the characteristics of the study population are summarized in Table 1.

### Relationship between TMAO and butyrate with AUC-H2

**Figure 1:**
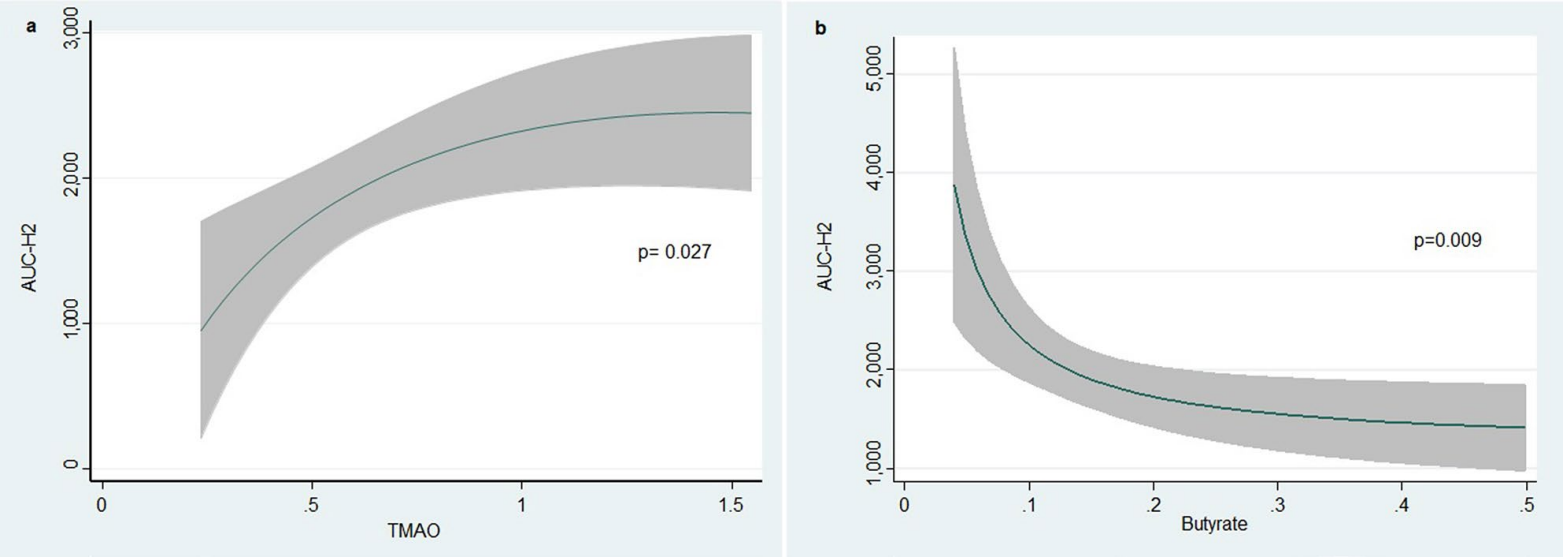
Multivariate association between AUC-H2 and blood bacterial metabolites (TMAO and Butyrate). *TMAO* Trimethylamine N-oxide, *AUC-H2* Area under the curve of hydrogen concentration.

**Figure 2:**
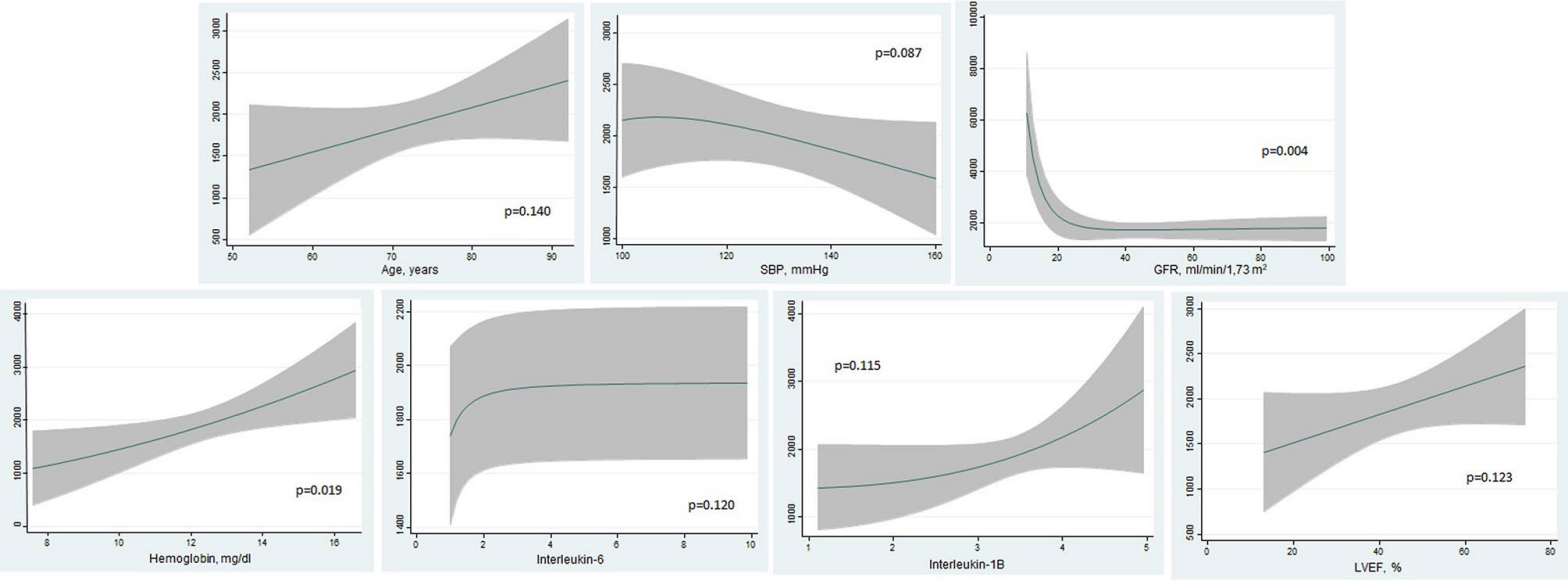
Direction and magnitude of the association among the covariates included in the multivariate Model 2 and AUC-H2. *AUC-H2* Area under the curve of hydrogen concentration, *SBP* Systolic blood pressure, *GFR* Glomerular filtration rate, *LVEF* Left ventricular ejection fraction.

In univariate analysis, TMAO and butyrate were significantly associated with AUC-H2 (p = 0.041 and p = 0.040, respectively). For TMAO, this association was positive and negative for butyrate (Supplementary File 1). Estimates of risk, adjusted for age and sex, showed similar significant findings (p = 0.006 and p = 0.003, for TMAO and butyrate, respectively) (Supplementary File 2). After a more thoroughly multivariate adjustment (Model 2), these associations remained significant (p = 0.027 and p = 0.009 for TMAO and butyrate, respectively). In this second model, TMAO was positive and non-linearly associated with higher AUC-H2 with a sharp slope below the percentile 75% of the distribution and a plateau afterward (p = 0.027) (Fig. 1a). On the other hand, butyrate showed an inverse non-linear association with AUC-H2 (p = 0.009) (Fig. 1b). Figure 2 showed the association among the covariate included in the final multivariate model 2 and the AUC-H2.

Using R^2^ as metric, the variables’ contribution for predicting the model in order of importance were: GFR (34.9%), interleukin-1β (12%), interleukin-6 (11.5%), butyrate (11.1%), TMAO (9.7%), hemoglobin (8.3%), SBP (5.1%), LVEF (4%), age (3.3%), and sex (< 0.01%) (Drop-in R2). The TMAO and butyrate models accounted for 60.5% and 45.7% of the variability in AUC-H2.

## Discussion

The potential role of the gut microbiota in the pathogenesis of metabolic and inflammatory disturbances in HF has been partially unveiled. In the current work, higher TMAO and lower butyrate identified a subset of patients with higher exhaled hydrogen after a lactulose breath test. Current findings suggest both metabolites disturbances share a common pathophysiological pathway with SIBO in patients with a recent episode of acute HF.

TMAO is generated from dietary nutrients such as phosphatidylcholine, choline, and L-carnitine (usually found in high-fat foods). Once ingested, multiple gut microbial enzyme complexes transform these molecules and their products circulate by the portal vein to the liver, where the flavin-containing monooxygenases produce TMAO^1,2^. In HF, TMAO is associated with greater HF-severity and worse prognosis^6–8^. On the other side, butyrate belongs to the short-chain fatty acids (SCFAs) family. They are mainly produced in the colon by bacterial fermentation of dietary fiber^2,12^. Loss of butyrate-producing bacteria may result in a dysfunctional intestinal mucosal barrier and leakage of microbial products such as lipopolysaccharide (LPS) that activate the innate immune system triggering inflammation^2,3^. Recent sequencing-based studies in HF patients have reported a relative reduction in taxa from the *Lachnospiracea or Ruminococcacea* families, species known for their capacity for butyrate production^2,12,13^. Interestingly, depletion of the known butyrate producer *Eubacterium Halli* and *Lachnospiracea* are associated with increased plasma levels of soluble CD25, more severe disease, and a higher risk of death or heart transplantation^13^.

In summary, our study shows that TMAO and butyrate levels in patients with HF may have a role in identifying those patients with a higher risk of gut microbiota derangements linked to the activation of deleterious metabolic and inflammation pathways. These findings endorse the abdominal and dysbiosis contribution in the pathophysiology of HF.

Important limitations deserved to be acknowledged. First, this is an observational, small, and single-center study. Second, the exhaled breath-tests are not considered the gold-standard for SIBO diagnosis; however, there is evidence showing an acceptable accuracy level^14^. Third, we did not register the type of dietary regimen, or the drugs consumed that may largely influence both metabolites' values. Finally, the clinical implications of these findings need to be settled with bigger studies. For instance, further studies should: (a) explore the potential utility of higher TMAO and lower butyrate for identifying an underlying SIBO in patients with a recent episode of acute HF and other pathologies, (b) confirm the causal contribution of SIBO for explaining metabolites disturbances in the clinical setting.

## Conclusions

Bacterial-origin metabolites TMAO and butyrate were independently related to AUC-H2 in patients with a recent hospitalization for acute HF. Further studies are needed to confirm the current findings and explore the clinical implication of them.

## Supplementary information

Supplementary information.
