# Supplementary material for: Bacterial metabolites trimethylamine N-oxide and butyrate as surrogates of small intestinal bacterial overgrowth in patients with a recent decompensated heart failure
Source: Sci Rep. 2021 Mar 17;11:6110. doi: 10.1038/s41598-021-85527-5 (PMC7969616; doi:10.1038/s41598-021-85527-5)
Supplement: Supplementary file 1 — Supplementary information. [file 41598_2021_85527_MOESM1_ESM.pdf]

Bacterial Metabolites Trimethylamine N-Oxide and Butyrate as Surrogates of Small Intestinal Bacterial Overgrowth in Patients with a Recent Decompensated Heart Failure

Authors:

Anna Mollar, PhD [a,b], Vannina G. Marrachelli, PhD [c,d,], Eduardo Núñez , MD, MPH[a], Daniel Monleon, PhD [c,d], Vicent Bodí, MD [a], PhD, Juan Sanchis MD, PhD [a, b], David Navarro MD, PhD[e], and Julio Núñez MD, PhD [a, b]

Affiliations:

[a] Cardiology Department. Hospital Clínico Universitario, INCLIVA. Universitat de València. Valencia, Spain.

[b] CIBER Cardiovascular, Madrid, Spain

[c] Metabolomic and Molecular Image Lab, Health Research Institute, INCLIVA, Valencia, Spain.

[d] Physiology Department Universitat de Valencia, Spain

e Microbiology Department. Hospital Clínico Universitario, INCLIVA. Universitat de València. Valencia, Spain.

Supplementary File 1:

Supplementary figure 1: AUC-H2 and blood bacterial metabolites TMAO and Butyrate. Univariate association.

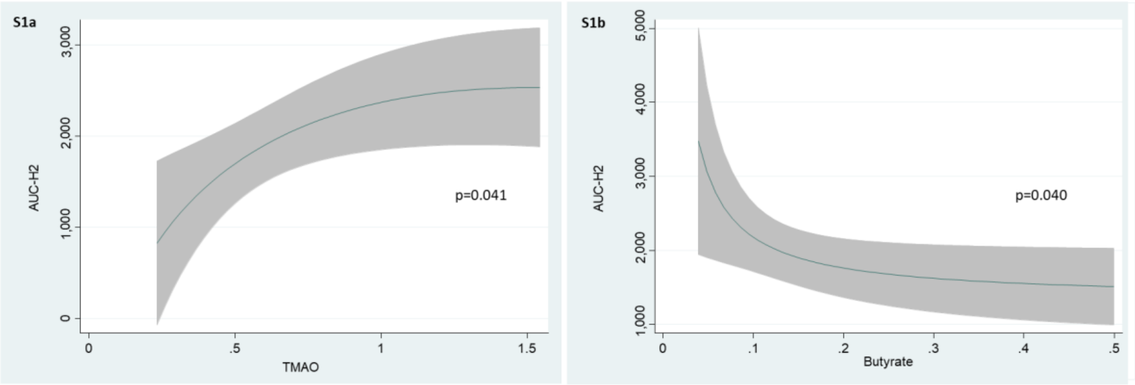

TMAO: Trimethylamine N-oxide; AUC-H2: Area under the curve of hydrogen concentration.

Supplementary File 2:

Supplementary figure 2: AUC-H2 and blood bacterial metabolites TMAO and butyrate adjusted for age and sex (Model 1).

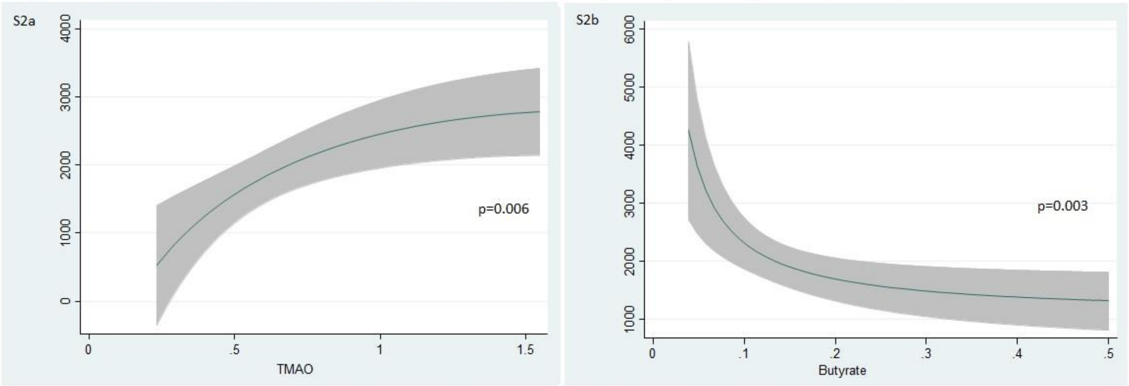

TMAO: Trimethylamine N-Oxide ; AUC-H2: Area under the curve of hydrogen concentration.
